# Graphene-based magnetic covalent organic frameworks and deep eutectic solvent functionalized adsorbents for polycyclic aromatic hydrocarbons: a review

**DOI:** 10.1098/rsos.251102

**Published:** 2025-10-08

**Authors:** Nurul Imanina Zameran, Noorashikin Md Saleh, Nur Hidayatul Nazirah

**Affiliations:** ^1^ Department of Chemical and Process Engineering, Faculty of Engineering and Built Environment, Universiti Kebangsaan Malaysia, Bangi, Selangor, Malaysia

**Keywords:** adsorption, polycyclic aromatic hydrocarbons, graphene-based magnetic covalent organic frameworks, deep eutectic solvents, sustainable adsorbent

## Abstract

Polycyclic aromatic hydrocarbons (PAHs) are hazardous organic compounds defined by their long-lasting nature, carcinogenicity and mutagenicity, posing environmental and health risks. Conventional PAH removal methods usually have high costs, low efficiency and potentially produce secondary contaminants. Graphene-based magnetic covalent organic frameworks (MCOFs) emerged as an alternative, offering high surface area, tuneable functionality and superior magnetic separability. Graphene-based MCOFs facilitate strong π–π interactions, hydrophobic effects and improved adsorbent stability, leading to more efficient PAH removal. The distinctive focus of this review is on deep eutectic solvents (DESs) as a green modification strategy to further optimize adsorption efficiency by lowering reaction temperature, reducing synthesis time and eliminating the use of hazardous organic solvents. This review presents a comprehensive study of graphene-based MCOFs and DES functionalized adsorbent synthesis, adsorption mechanisms and their environmental applications in enhancing PAH adsorption performance as the next-generation sustainable materials for large-scale PAH remediation. Collectively, these advances align with the principles of green chemistry and are anticipated to contribute to global efforts towards the sixth, seventh and fourteenth sustainable development goals, namely clean water and sanitation, affordable and clean energy and the protection of aquatic ecosystems.

## 1. Introduction

The global water crisis is exacerbated by both resource scarcity and increasing contamination, posing serious environmental and public health risks. Among the most persistent and hazardous contaminants are polycyclic aromatic hydrocarbons (PAHs), a subclass of persistent organic pollutants (POPs) recognized by the Stockholm Convention for their longevity, resistance to degradation, bioaccumulation potential and toxicity [1]. PAHs originate from natural processes, such as wildfires and volcanic activity, as well as anthropogenic sources including fossil fuel combustion, industrial discharges, traffic emissions and domestic activities [2,3]. Their widespread presence across air, water, soil, biota, and even in remote ecosystems, underscores their environmental persistence [4,5].

Human exposure to PAHs occurs via ingestion, inhalation and dermal contact, with adverse effects ranging from endocrine disruption to carcinogenesis, depending on the exposure level, duration and compound toxicity [6,7]. Conventional remediation methods, such as thermal, chemical and biological treatments, often fall short because of operational complexity, high costs and the generation of harmful by-products [8,9]. As such, adsorption has emerged as a preferred alternative for PAH removal owing to its cost-effectiveness, operational simplicity and environmental compatibility [10,11].

Advanced nanomaterials have significantly enhanced adsorption-based remediation. Magnetic nanoparticles (MNPs), particularly Fe_3_O_4_ and γ-Fe_3_O_4_, offer high surface area, reusability and ease of separation, but suffer from aggregation and oxidation without surface modification [12,13]. Covalent organic frameworks (COFs) promote another promising class of porous crystalline materials, offering high surface area, thermal stability and tuneable porosity. However, their powdery form complicates recovery and reuse [14]. The integration of COFs with MNPs has led to the development of magnetic covalent organic frameworks (MCOFs), which combine high adsorption capacity with magnetic separability, structural stability and enhanced π–π interactions [15].

Despite their promise, conventional COF synthesis methods such as solvothermal, mechanochemical and ionothermal techniques still require high energy input, extended reaction times or toxic solvents [16]. Deep eutectic solvents (DESs), formed by mixing a hydrogen bond donor and acceptor, have emerged as green alternatives to traditional solvents, offering low toxicity, biodegradability, thermal stability and tuneable solvation properties [17,18]. Recent studies prove that DESs can be used both in the synthesis process of COFs and to modify magnetic adsorbents, making them more water-repellent and better at capturing PAHs (19; [20]).

Graphene oxide (GO), with its large surface area, chemical stability and π–electron system, is frequently used in adsorbent fabrication. However, GO tends to aggregate via π–π stacking, reducing adsorption efficiency. Its magnetic derivative, magnetic graphene oxide (MGO), improves separation performance, but hydrophobicity properties remain limited [21] . Functionalization with DESs has proven that introducing hydrogen bonding sites improves hydrophobic interactions, thereby enhancing PAH adsorption capacity [19].

Magnetic solid-phase extraction (MSPE) has emerged as a powerful technique for the rapid, simple and efficient isolation of contaminants from complex matrices. Its performance is largely governed by the properties of the magnetic sorbent, which can be optimized through surface functionalization with polymers, surfactants or framework materials [22–24]. The large surface area contacts between sorbents and analytes, combined with magnetic field-assisted separation, significantly enhance extraction efficiency [25,26]. MSPE has been extensively employed for the detection of environmental pollutants in water, as demonstrated by numerous studies [27].

**Figure 1.**
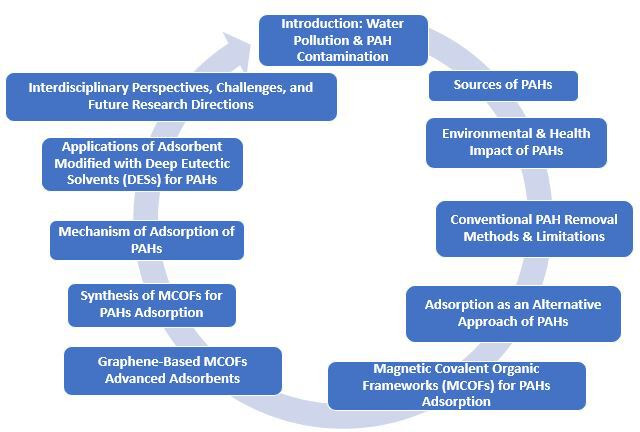
Overview of this review paper.

This review presents recent advances in MCOFs, their synthesis, adsorption mechanisms and environmental applications in PAH removal, with a focus on graphene-based MCOFs as an efficient adsorbent for PAH pollutants (figure 1). Furthermore, it highlights the novelty of the use of DESs as a functionalization strategy to further enhance PAH removal efficiency, offering a green, innovative and sustainable approach in the production of adsorbents. By addressing current progress and challenges, this work underscores the potential of magnetic graphene-based COF nanomaterials and DES-modified adsorbents as next-generation adsorbents for large-scale and sustainable PAH removal that align with green chemistry principles and contribute to fulfilling the sixth, seventh and fourteenth sustainable development goals (SDGs), which are to have access to clean water and sanitation, to have affordable and clean energy and to sustain life below water.

## 2. Overview of polycyclic aromatic hydrocarbons

PAHs are a class of POPs that pose significant environmental and health hazards. These complex organic compounds consist of carbon and hydrogen atoms arranged in fused ring structures containing at least two benzene rings [28]. Owing to their toxicological significance, PAHs have been classified as priority pollutants by the European Union (EU), the Environmental Protection Agency (EPA) and the International Agency for Research on Cancer. Many PAH compounds and their derivatives exhibit carcinogenic, mutagenic, teratogenic and genotoxic properties, with well-documented links to oxidative stress, diabetes, obesity, cardiovascular diseases and cancer [29].

**Figure 2.**
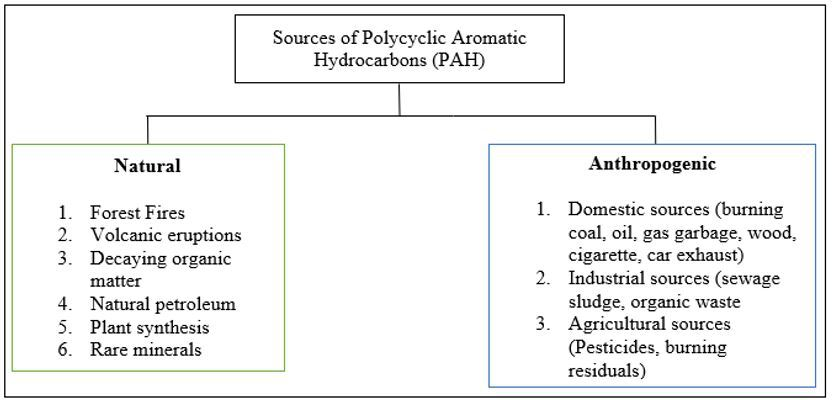
PAH sources (adapted from [30]).

PAHs originate from both natural and anthropogenic sources, as shown in figure 2, largely influenced by urbanization and industrialization [31]. The predominant sources of PAHs are pyrogenic, arising from the incomplete combustion of organic materials, including fossil fuels, petroleum products and biomass [32]. Other significant contributors include volcanic activity, coal mining, transportation emissions, power plants and accidental petroleum spills. Additionally, PAHs of diagenetic or biogenic origin are derived from plants, algae, microbes, phytoplankton or through the gradual transformation of organic matter over time [33].

The physicochemical traits of PAHs strongly influence their environmental transformation and stability. These compounds are highly hydrophobic and lipophilic, rendering them resistant to biodegradation [34]. They are characterized by low water solubility, moderate volatility and high melting and boiling points. PAH solubility decreases as the number of aromatic rings increases, affecting their transport and bioavailability in aquatic ecosystems.

Low molecular weight PAHs (fewer than three benzene rings) are relatively more soluble in water and undergo rapid decomposition through dissolution, volatilization and microbial degradation. These compounds are acutely toxic to aquatic organisms, particularly fishes [35]. By contrast, PAHs containing three to five benzene rings exhibit lower water solubility, leading to their accumulation in aquatic organisms and adherence to solid particulates. High molecular weight PAHs (more than five benzene rings) exhibit extreme persistence, with degradation rates so slow that they can remain in sediments for years [36]. PAHs tend to adhere to aquatic sediment particles, posing severe risks to benthic organisms and potentially bioaccumulating through the biological chain, ultimately impacting living organisms [37,38]. Owing to their mutagenic and carcinogenic potential, prolonged exposure to PAHs has been associated with an increased risk of cancers, including those of the bladder, skin and lungs [39–41].

Human exposure to PAHs occurs primarily through three main routes: inhalation of contaminated air, ingestion of polluted food and water and dermal contact [42,43]. These harmful substances have been associated with multiple health impacts, such as respiratory issues, skin irritations and chronic diseases [44]. Given the severe environmental and health implications of PAH contamination, the development and implementation of effective remediation strategies remain an urgent priority. Given the significant health risks associated with exposure to PAHs, various international regulatory agencies have established strict guidelines for their concentrations in aquatic environments (table 1). These regulations commonly use benzo[a]pyrene (BaP) as a reference compound owing to its high carcinogenicity.

**Table 1.** Guidelines and regulation on PAHs in the environment by different regulatory agencies. (BaP, benzo[a]pyrene; BbF, benzo[b]fluoranthene; BkF, benzo[k]fluoranthene; BghiP, benzo[ghi]perylene; IcdP, indeno[1,2,3-cd]pyrene; Ant, anthracene; Flu, fluorene; Nap, naphthalene; BaA, benzo(a)anthracene; DahA, dibenzo(a,h)anthracene)

agency	environmental media	compound	established standard (µg l^−1^)
EU	surface waters ([45], 2013/39/EU)	PAHs	0.27
		BaP	0.027
		BbF	0.017
		BkF	0.017
		BghiP	8.2 × 10^–4^
		IcdP	—
		NAP	130
		ANT	0.12
		FLE	0.1
EU	drinking water (Directive 98/83/EC, [46])	BaP	0.01
		PAHs	0.10 (sum of BbF, BkF, BghiP and IcdP)
WHO	drinking water [47]	BaP	0.7
US EPA	ambient water [48,49]	BaA	0.0012
		BaP	0.00012
		BbF	0.0012
		BkF	0.012
		DahA	0.00012
		IcdP	0.0012
US EPA	drinking water [50]	BaP	0.2
France	discharges into environment [51]	Ant	25
		Flu	25
		Nap	130
		PAHs	25 (sum of BaP, BbF, BkF, BghiP and IcdP

In the EU, PAHs have been regulated for decades under frameworks such as the Water Framework Directive ([52], last consolidated in 2014), which classifies PAHs as priority substances in surface waters. Environmental Quality Standards (EQS) for PAHs, including BaP, benzo[b]fluoranthene (BbF), benzo[k]fluoranthene (BkF), benzo[ghi]perylene (BghiP) and indeno[1,2,3-cd]pyrene (IcdP), are detailed in [45], as amended by Directive 2013/39/EU. Additionally, EQS values are also established for naphthalene, anthracene and fluorene. In drinking water, the EU Drinking Water Directive (Directive 98/83/EC, amended by [46]) mandates a maximum concentration of 0.10 µg l^−1^ for the sum of four PAHs (BbF, BkF, BghiP and IcdP), and a more stringent limit of 0.01 µg l^−1^ for BaP alone, which is also used as a tracer for total PAH contamination.

The World Health Organization (WHO) [47] recommends a guideline value of 0.7 µg l^−1^ for BaP in drinking water, while the United States (US) EPA enforces more stringent limits, with ambient water criteria ranging from 0.00012 to 0.012 µg l^−1^ depending on the PAH, and a maximum allowable BaP concentration of 0.2 µg l^−1^ in drinking water. France has established its own regulations through the Ministerial Order of 2 February 1998 [51] (last amended in 2023), setting discharge limits for multiple PAHs, including ANT, fluorene (FLE), NAP and the five high-priority PAHs into natural water bodies.

These regulatory values vary significantly across jurisdictions. For instance, while the WHO and US EPA allow higher BaP concentrations (0.7 and 0.2 µg l^−1^, respectively), the EU’s much lower limit of 0.01 µg l^−1^ reflects a more precautionary approach. This EU threshold is 100 times lower than BaP’s water solubility, indicating that such concentrations are environmentally realistic and routinely encountered. Moreover, regulatory limits for industrial discharges can reach up to 25 µg l^−1^ (or 130 µg l^−1^ for NAP), highlighting the potential for significant environmental contamination if not properly managed.

### 2.1. 16 Polycyclic aromatic hydrocarbons

**Figure 3.**
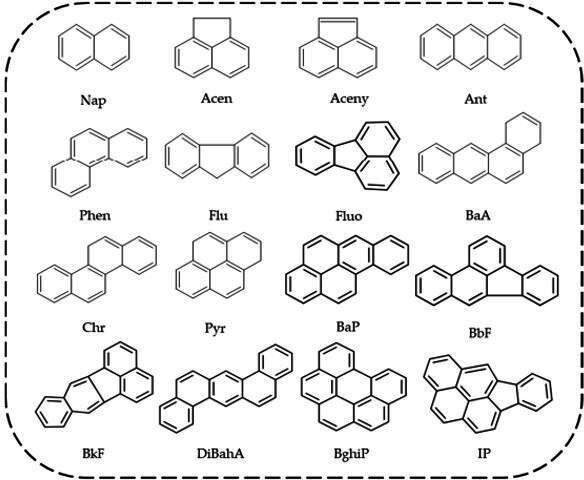
Structure of the 16 US EPA PAHs [53].

The US EPA has identified 16 PAHs as priority contaminants because of their significant potential for human exposure, toxicity, widespread prevalence at hazardous waste sites and the availability of extensive toxicological and environmental data. These priority PAHs include Naphthalene (Nap), Acenaphthene (Acen), Acenaphthylene (Aceny), Anthracene (Ant), Phenanthrene (Phen), Fluorene (Flu), Fluoranthene (Fluo), Benz[a]anthracene (BaA), Chrysene (Chr), Pyrene (Pyr), Benzo[a]pyrene (BaP), Benzo[b]fluoranthene (BbF), Benzo[k]fluoranthene (BkF), Dibenzo[a,h]anthracene (DiBahA), Benzo[g,h,i]perylene (BghiP), and Indeno[1,2,3-cd]pyrene (IP) as shown in figure 3. These compounds are of particular concern because of their potential for human exposure, toxicity, prevalence at hazardous waste sites and the availability of information [54]. Given their hazardous nature, regulatory agencies prioritize monitoring, controlling and mitigating their impact on both environmental and public health.

### 2.2. Substituted polycyclic aromatic hydrocarbons

Current research on PAHs has predominantly focused on homocyclic molecules. However, approximately some of the identified aromatic compounds are heterocyclic, containing sulfur, oxygen and/or nitrogen as ring substituents on one or more carbon atoms. These substituted PAHs exhibit distinct physicochemical properties that influence their environmental behaviour and toxicity.

Once released into the atmosphere, PAHs can undergo chemical transformations through interactions with atmospheric oxidants such as nitrogen oxides (NOx), ozone (O_3_) and hydroxyl radicals (OH), leading to the formation of substituted PAHs [55]. These compounds may subsequently enter water ecosystems either directly from airborne particulate matter or through precipitation fallout [56].

Nitrated polycyclic aromatic hydrocarbons (NPAHs) are usually detected alongside PAHs in various environmental compartments. Although typically present in lower concentrations compared with their parent PAHs, NPAHs can exhibit significant toxic effects [57]. The introduction of a nitrogen atom into the PAH structure increases the compound’s polarity and enhances its solubility in water, which influences its environmental distribution and potential impact on aquatic organisms. Another category of PAHs, oxygenated polycyclic aromatic hydrocarbons (OPAHs), are hazardous chemical compounds commonly found in the atmosphere. Owing to their semi-volatile nature and reduced vapour pressures compared with their parent PAHs, OPAHs tend to adsorb onto airborne particulate matter [58].

As urbanization accelerates, natural landscapes such as forests and undeveloped land are increasingly replaced by impervious surfaces, including roads, parking lots and residential driveways. This transition intensifies surface runoff during rainfall and snowmelt events, facilitating the transport of substituted PAHs such as sulfur-containing PAHs into urban streams [59]. The accumulation of these pollutants in aquatic systems underscores the need for effective mitigation strategies to minimize their environmental and human health risks.

## 3. Adsorption

**Figure 4.**
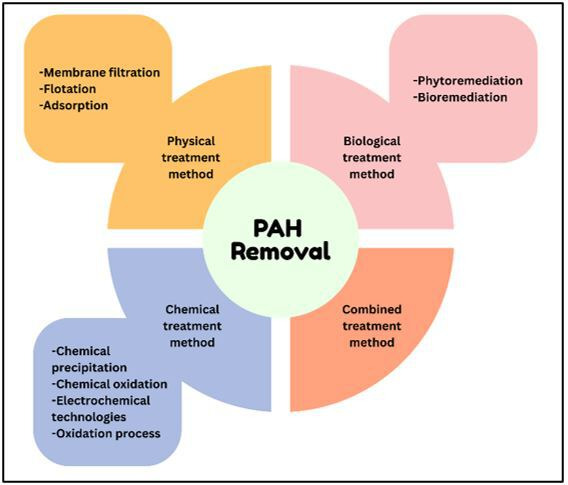
Methods for the removal of PAHs.

Various physical, chemical, biological and combined treatment steps have been employed to remove PAHs from the environment [60]. Common techniques include incineration, photolysis and thermal desorption [61], chemical oxidation [62], zero-valent iron treatment [63], combined ultrasound and zero-valent iron/ethylene diamine tetraacetic acid (EDTA) processes [64], electrochemical techniques [65], biodegradation [66], as well as advanced oxidation processes, bioremediation, membrane separation, coagulation-flocculation and adsorptive processes [67] (figure 4). However, many of these approaches are impractical for large-scale applications owing to challenges such as high reagent requirements, prolonged treatment durations, specific pH conditions and substantial investment and maintenance costs [64]. Among these, adsorption has gained considerable attention because of its high selectivity, cost-effectiveness, ease of adsorbent recovery and minimal waste production compared with other treatment procedures [68–70].

Adsorption is a surface-based process influenced by the adsorbent material’s porous structure and chemical properties. As molecules bind to the adsorbent surface, their separation from the fluid phase depends on molar mass, shape and polarity. Based on the interaction between adsorbate and adsorbent, adsorption is classified into physical adsorption (physisorption) and chemical adsorption (chemisorption). Physisorption is a reversible process occurring at low temperatures, where weak electrostatic interactions such as Van der Waals forces, dipole–dipole interactions, π–π interactions and hydrogen bonding govern the binding [71]. By contrast, chemisorption is an irreversible process that occurs at higher temperatures, involving electron transfer between the adsorbent surface and the adsorbate, altering the adsorbate’s chemical nature [72,73]. Adsorption has been widely used for wastewater treatment, effectively removing various contaminants such as organic pollutants, heavy metals, dyes, nutrients and pathogens [74].

**Figure 5.**
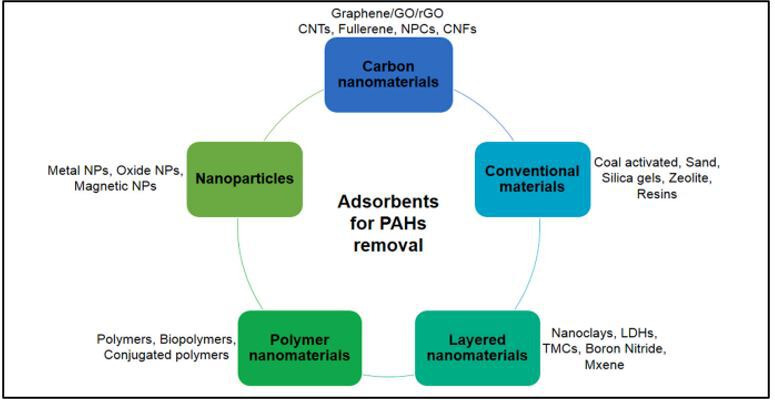
Types of adsorbents used for PAH adsorption [75].

Several adsorbents (figure 5) employed for PAH removal include activated carbon [76], bentonite [77], biochar [78], chitosan [79], graphene [80], carbon nanotubes [81] and zeolite [82]. The origin of adsorption technology was in the late nineteenth century when R.V. Ostrejko patented the first activated carbon filter in 1900 for wastewater treatment [83]. Since then, activated carbon has been widely used because of its high surface area, porosity and strong affinity for organic pollutants. However, it also has drawbacks, such as high cost, limited selectivity and challenges in regeneration [84]. The increasing complexity of wastewater and stricter environmental regulations have driven the search for more efficient, cost-saving and sustainable adsorbents beyond traditional materials [83]. The advent of nanotechnology has revolutionized adsorbent synthesis, enabling modifications at the molecular level to enhance pollutant capture efficiency. Innovative materials such as carbon nanotubes, graphene, metal-organic frameworks (MOFs) and nanostructured polymers have demonstrated superior performance, selectivity and reusability compared with conventional adsorbents. These nanomaterials exhibit remarkable capabilities in removing a variety range of contaminants, including heavy metals, organic pollutants, pharmaceuticals and endocrine disruptors, setting new standards for water purification technology [85].

## 4. Functionalization of covalent organic frameworks in magnetic nanoparticles as an adsorbent for polycyclic aromatic hydrocarbons

### 4.1. Magnetic nanoparticles in adsorption

MNPs are popular for their controlled particle size, low cost, easy preparation and superparamagnetism properties. A significant benefit of MNPs is their superparamagnetic characteristic, enabling easy manipulation by magnetic field gradients [86]. These unique properties enable applications of MNPs in biomedical imaging, drug administration, hyperthermia therapy and magnetic separation processes. MNPs based on iron oxides, such as γ-Fe_2_O_3_ and Fe_3_O_4_, are commonly employed owing to their excellent stability, availability, simple production and biocompatibility [12]. Pure metal particles, such as Fe and Co particles, or other forms of MNPs, such as NiFe_2_O_4_, CoFe_2_O_4_ and CuFe_2_O_4_, which contain a lower amount of Co, Ni, Cu, Zn or Mn, were used in some circumstances to increase saturation magnetization [87].

However, these heteroatom-doped MNPs have several limitations, such as cobalt and nickel, which are highly poisonous and can be oxidized easily. As a result, just a few investigations of their application have been reported [88]. Additionally, MNPs have several drawbacks, including a propensity to agglomerate in water-based conditions because of Van der Waals forces, which hinders direct application, and they are prone to oxidation upon exposure to oxygen [13,89]. To address these restrictions, the MNP surfaces must be covered with suitable fabricating agents. Functionalized MNPs are engineered to efficiently remediate wastewater by adsorbing targeted pollutants [90]. The surface modification of MNPs using COFs enables the synthesis of magnetite nanoparticles combined with COFs (MCOFs), which function as novel adsorbents currently under preliminary investigation.

### 4.2. Covalent organic frameworks as adsorbents

Over the past decade, adsorbents have been improved in their adsorption capacities and specificities, adsorption mechanisms and kinetics. Nanotechnology has revolutionized adsorbent production by creating nanoscale adsorbent materials with distinct features such as high surface area, variable pore size and shape and functional groups. Nanomaterials can act as adsorbents or as building blocks for composites [91].

The properties of porous materials, including zeolites, activated carbons, MOFs and COFs, attract researchers owing to their low density, large specific surface area and tailored pore structure, as advantageous for adsorption, separation, sensing, energy, gas storage and elimination processes [15]. COFs are a category of porous crystalline structures composed of light components (C, H, B, N, O, etc.) interconnected by robust covalent bonds. These materials exhibit numerous intriguing characteristics, including elevated specific surface area, exceptional thermal stability, high porosity and low density [14]. COFs are innovative chemical molecules attracting significant interest from researchers. Nonetheless, COFs are usually found in powdered form, leading to challenges in their operation and recycling, as well as the possible risk of secondary pollution. Thus, because of these limitations, researchers have functionalized COFs with MNPs.

### 4.3. Magnetic covalent organic frameworks: a hybrid adsorbent

Combining COFs with magnetic techniques to create MCOF composites has gained interest for its potential to meet application needs and drive further research. MCOFs represent another novel category of materials that serve as highly efficient magnetic adsorbents, especially in environmental analysis. Their remarkable attributes include unique morphology, economical synthesis, chemical and thermal stability, extensive specific surface area, substantial adsorption capacity, robust π–π interactions with analytes, uniform pore size, excellent reusability and ease of magnetic recovery [15].

In recent years, the integration of COFs with magnetic techniques to produce MCOFs, which possess the advantages of both, has garnered considerable interest in fulfilling application demands and advancing further improvements. Their unique attributes, such as a well-developed pore structure, less aggregation of MNPs and enhanced magnetic responsiveness, confer exceptional adsorption capacity and remarkable separation capability. The advantages of facile separation and recovery of MCOF materials post-adsorption provide them with an intriguing alternative platform, enhancing manageability and recyclability. The unique advantages render the investigation of novel MCOFs a potential approach in environmental remediation, especially in the adsorptive elimination of hazardous contaminants [92].

### 4.4. Application of magnetic covalent organic frameworks in polycyclic aromatic hydrocarbon adsorption

Currently, MCOFs have been rapidly synthesized to be used as an adsorbent to remove pollutant PAHs. The MCOFs adsorbent in the application of PAHs adsorption are listed in table 2.

**Table 2.** Application of MCOFs in PAH adsorption. (Sulfur-containing heterocyclic aromatic hydrocarbons (PASHs), polychlorinated naphthalenes (PCNs), atmospheric pressure gas chromatography tandem mass spectrometry (APGC-MS/MS), high performance liquid chromatography (HPLC), fluorescence detection (FLD), diode-array detection (DAD), dispersive solid-phase extraction (dSPE)).

adsorbent	samples	PAHs	detection method	adsorbent mass (mg)	adsorption time (min)	relative recoveries (%)	limit of detection	reusability absorbent	reference
COF-LZU1@PEI@Fe_3_O_4_	water and soil	6	MSPE, HPLC-FLD	5	30	85.1−107.8	0.2−20 pg ml^−1^	6 times	[14]
magnetic bouquet-shaped COF (TpPa−1)	water	6	MSPE, HPLC-FLD	5	—	73−110	0.24−1.01 ng l^−1^	—	[93]
Fe_3_O_4_@COF-(TpBD)	food	15	MSPE, HPLC-DAD	10	12	84.3−107.1	0.83−11.7 ng l^−1^	3 times	[94]
Fe_3_O_4_@COF(TpDA)	food	15	MSPE, HPLC-DAD	10	10	85.5−104.2	0.03−0.73 µg l^−1^	6 times	[95]
Fe_3_O_4_@COFeCOOH	water	13 PAHs, TCs, TDs	MSPE, HPLC-DAD	10	10	93.6−105.8	0.003−0.008, 0.02−0.06, and 0.006−0.008 mg l^−1^	15 times	[96]
Fe_3_O_4_@SiO_2_@COF-`	water	16	d-SPE, GC-MS	2	3	83−116	1.5−15.1 ng l^−1^	—	[97]
Fe_3_O_4_@TAPT-DMTA-COF	food	5 N-PAHs	MSPE, GC–MS/MS	12.5	10	80.26−90.81	1.43−17.24 ng l^−1^	—	[98]
Fe_3_O_4_@TPPCl_4_	air	8 PCNs	MSPE, APGC-MS/MS	0.8	15	93.11−105.81	0.005−0.325 ng l^−1^	8 times	[99]
Fe_3_O_4_@COF(Tp-NDA)	food	10	MSPE, HPLC-UV	2	3	74.6−101.8	0.05−0.25 µg l^−1^	10 times	[29]
Fe_3_O_4_/COF-DQTp	water	1	MSPE, HPLC-FLD	19	10	—	—	2 times, adsorption performance (>90%) 4 times, adsorption capacity (reduced to 80%)	[100]
Fe^0^ @MXene@COF-LZU1	food	4 PAHs and 2 PASHs	MSPE, GC-MS/MS	70	60	83.56 −93.40	0.014−0.83 ng l^−1^	—	[101]

The development of MCOF adsorbents has significantly enhanced PAH analysis for environmental and food samples. Various studies have introduced novel MCOFs with distinct structural modifications, leading to improved adsorption efficiency, recovery rates and reusability. This review provides a comparative analysis of different MCOF adsorbents, emphasizing their performance metrics and advancements over time. Wang & Chen [14] synthesized COF-LZU1@PEI@Fe₃O₄ by covalently immobilizing COF-LZU1 onto polyethyleneimine-functionalized MNPs. The adsorbent demonstrated high extraction efficiency for PAHs in water (90.9−107.8%) and soil (85.1−105.0%), establishing a benchmark for subsequent MCOF designs. This work laid the foundation for using MCOFs in environmental sample analysis, showing that they could achieve high recovery rates and reliable performance. Similarly, He *et al*. [93] developed a bouquet-shaped MCOF (TpPa-1@Fe₃O₄), which exhibited enhanced specific surface area and superparamagnetic properties, making it ideal for trace PAH extraction via MSPE. The unique bouquet-like morphology increased the available adsorption sites, leading to better PAH capture and improved analytical performance.

Building upon these designs, Li *et al*. [94] introduced Fe₃O₄@COF(TpBD), a core-shell nanostructured hybrid microsphere with superior enrichment efficiency (99.95%) and rapid adsorption kinetics (12 min equilibrium). The rapid adsorption time is a significant advantage, as it reduces the duration of sample preparation while maintaining high sensitivity. Shi *et al*. [95] further enhanced adsorption performance with Fe₃O₄@COF(TpDA), specifically designed for PAH detection in edible oils and grilled meats. The material’s high porosity and strong affinity for PAHs allowed for precise and efficient extraction in complex food matrices. Hu *et al*. [96] advanced MCOF technology by creating Fe₃O₄@COFeCOOH, capable of adsorbing multiple analytes (PAHs, tetracyclines and triphenylmethane dyes) and demonstrating remarkable reusability over 15 cycles. This multifunctional capability marks a significant step forward in analytical chemistry, allowing a single adsorbent to handle diverse pollutants efficiently.

Yu *et al*. [97] introduced a three-dimensional MCOF (Fe₃O₄@SiO₂@COF-300) synthesized via a layer-by-layer approach. This adsorbent showcased outstanding structural stability, precision in intra-day and inter-day analyses and a low detection limit, proving its effectiveness for 16 PAHs. The integration of computational methods like density functional theory calculations further strengthened the understanding of its adsorption mechanisms, enhancing its credibility as a high-performance adsorbent. Wang *et al.* [98] then developed Fe₃O₄@TAPT-DMTA-COF, featuring a grapevine structure with high surface area (1578.45 m² g^−1^), strong magnetism and electronegative triazine groups for enhanced nitrogen-containing PAH (N-PAH) detection. The presence of triazine groups enhances interactions with N-PAHs, making this material particularly useful for detecting complex nitrogenous pollutants in environmental and food samples. Guo *et al*. [99] introduced Fe₃O₄@TPPCl₄ for polychlorinated naphthalenes (PCNs) detection, achieving high enrichment factors, an extensive linear range and low limits of detection. This adsorbent’s strong selectivity for PCNs, owing to its chlorine-rich structure, makes it highly effective for detecting and removing these persistent organic pollutants. Wu *et al*. [29] synthesized Fe₃O₄@COF(Tp-NDA) using a solvothermal method, demonstrating strong adsorption efficiency for PAHs in lake water, tea, coffee and fried chicken samples, with relative recoveries of 74.6−101.8% and stability for up to 10 cycles. The ability to maintain efficiency over multiple reuse cycles highlights its durability and cost-effectiveness, making it a practical option for real-world applications.

Pang *et al*. [100] designed Fe₃O₄/COF-DQTp, an MCOF adsorbent with 99% adsorption efficiency for BaP, achieving a high adsorption capacity of 19 milligram per gram only within 10 min and excellent reusability. The targeted approach in removing BaP, a known carcinogenic PAH, demonstrates the potential for MCOFs in public health and safety applications. More recently, Jiang *et al*. [101] introduced Fe₀@MXene@COF-LZU1, a ternary magnetic nanocomposite optimized for MSPE-gas chromatography-tandem mass spectrometry (MSPE-GC-MS/MS) detection of PAHs and sulfur-containing heterocyclic aromatic hydrocarbons (PASHs) in Chinese tea samples. COF-LZU1’s superior chemical stability in aqueous and organic solvents allows for its application across different sample types. This method provided advantages, such as rapid analysis, robustness, an extended linear range, reduced detection limits and excellent reproducibility, making it a highly effective alternative for trace-level PAH and PASH detection.

## 5. Synthesis of magnetic covalent organic frameworks as adsorbents of polycyclic aromatic hydrocarbons

**Figure 6.**
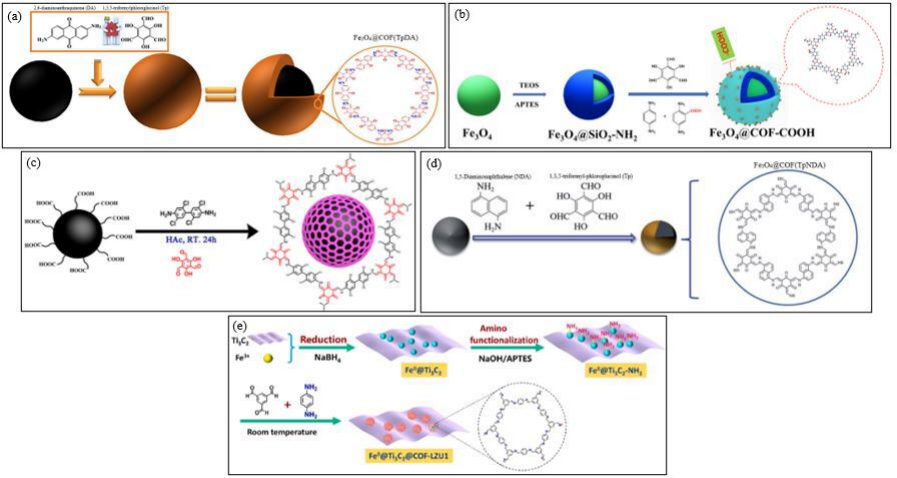
Schematic synthesis of (a) Fe_3_O_4_@COF(TpDA) [95], (b) Fe_3_O_4_@COF-COOH [96], (c) Fe_3_O_4_@TPPCl_4_ nanoparticles [99], (d) Fe_3_O_4_@COF(Tp-NDA) [29], and (e) Fe^0^@Ti_3_C_2_@COF-LZU1 [101].

MCOFs have emerged as highly effective adsorbents for the removal and detection of PAHs in various environmental and food matrices. These materials are synthesized through diverse methods, including aldehyde–amine condensation, Schiff base condensation, solvothermal processes and co-precipitation, which contribute to their high porosity, large surface areas and strong adsorption capacities. For instance, Shi *et al*. [95] (figure 6a) developed Fe_3_O_4_@COF(TpDA) via aldehyde–amine condensation of 1,3,5-triformylphloroglucinol (Tp) and 2,6-diaminoanthraquinone (DA) on Fe_3_O_4_ nanoparticles, yielding a material with supermagnetic properties and high efficiency in PAH removal from food samples. Similarly, Hu *et al.* [96] (figure 6b) synthesized Fe_3_O_4_@COFeCOOH using Schiff base condensation, enabling the concurrent adsorption of PAHs, tetracyclines (TCs) and triphenylmethane dyes (TDs) through hydrophobic, π–π interactions and weak cation exchange mechanisms. Expanding on the synthesis strategies, Yu *et al*. [97] applied a layer-by-layer method to fabricate Fe_3_O_4_@SiO_2_@COF-300, optimizing the coating thickness for enhanced adsorption efficiency. Meanwhile, Wang *et al*. [98] introduced Fe_3_O_4_@TAPT-DMTA-COF, which featured a crystalline grapevine structure with selective adsorption of N-PAHs because of its abundant carboxylate groups and highly ordered two-dimensional sheets.

Further innovations in MCOF synthesis were demonstrated by Guo *et al*. [99] (figure 6c), who developed Fe_3_O_4_@TPPCl_4_ through a one-pot approach at room temperature. This material exhibited exceptional thermal stability and was particularly suited for the analysis of PCNs in complex environmental samples. The solvothermal synthesis of Fe_3_O_4_@COF(Tp-NDA) by Wu *et al*. [29] (figure 6d) further underscored the adaptability of MCOFs, as their adsorbent efficiently extracted PAHs from various food and water samples, benefiting from π–π stacking and hydrophobic forces. The application of co-precipitation was highlighted in Pang *et al*. [100], where Fe_3_O_4_/COF-DQTp achieved a remarkable 99% adsorption efficiency for BaP in just 10 min, showcasing excellent stability and reusability. More recently, Jiang *et al*. ([101]; figure 6e) advanced the field by synthesizing Fe^0^@Ti_3_C_2_@COF-LZU1, a magnetic MXene-based COF, using electrostatic attraction and Schiff base reactions to create a highly durable and sensitive adsorbent for the detection of PAHs and PASHs in tea samples.

Collectively, these studies highlight the versatility and efficacy of MCOFs, demonstrating how their structural properties, functionalization strategies and magnetic characteristics contribute to enhanced adsorption capabilities. The incorporation of functional groups enables selective adsorption through π–π interactions, hydrophobic forces and electrostatic attractions, while robust magnetism allows for easy separation and regeneration. Furthermore, the reusability and stability of these materials underscore their potential for practical applications in pollution control and environmental monitoring, making them promising candidates for addressing contamination challenges in food safety and environmental protection.

## 6. Adsorption of polycyclic aromatic hydrocarbons using magnetic graphene nanoparticles

### 6.1. Magnetic graphene-based nanoparticles for polycyclic aromatic hydrocarbon adsorption

Raw MNPs could escape into a low pH medium and are prone to oxidizing in the ambient surroundings. On the other hand, MNPs are susceptible to aggregation in solutions because of their elevated surface energy and extensive specific surface area, hence diminishing their adsorption capability and constraining their applicability. MNPs are usually modified with different materials, including heteropoly acid, conductive polymers, Schiff base ligands, hemimicelles and carbon nanotubes, to improve performance [102,103]. Researchers have drawn interest in some graphene-based materials for their usage in the adsorption of PAHs from wastewater. The features of graphene-based materials, which consist of elevated surface area, mechanical durability and cost-effective production, facilitate their application in the adsorption treatment of effluents [104]. GO, characterized by an abundant number of oxygen-containing functional groups such as carboxyl, hydroxyl and epoxy groups, comprises one-atom-thick, two-dimensional layers of sp^2^-bonded carbon. Robust π–π stacking, hydrophobic interactions and hydrogen bonding facilitate interactions between the adsorbent and organic molecules [105–107]. By means of electrostatic interactions between GO sheets, negatively charged and the surface of Fe_3_O_4_ that have positively charged, GO can produce magnetic nanocomposites with magnetite, hence improving separation. The great adsorption capacity of GO is combined with the practicality of magnetic separation in the MGO nanocomposites. Moreover, different groups can be used to alter the hybrid material to prevent aggregation and increase its uses [108,109].

The integration of the magnetic characteristics of MNPs with the ideal surface features and nanoscale structures of GO is anticipated to generate MGO as a superior adsorbent. An alternative solution to the problem of MNP aggregation was found by Amiri *et al*. [110]. They used graphene or GO sheets as a support and spacer, and the MNPs were uniformly attached to the surface of GO through the interaction of the -COOH groups. This reduced MNP aggregation and limited their expansion. The MSPE approach uses absorbent materials with a strong capacity for capturing analytes, which can diminish interference from matrix materials and enhance experimental sensitivity and precision [111,112]. Consequently, the improvement of new and effective adsorbents is the key emphasis of MSPE technology. MGO-based nanomaterials, which have attracted attention for PAH adsorption owing to their great selectivity, ease of separation and remarkable adsorption capacity, are examples of new adsorbents made possible by technological and nanotechnological advancements. The summary of comparative adsorption of PAHs using magnetic graphene-based nanoparticles is presented in table 3.

**Table 3.** Summary comparison of the adsorption of PAHs using magnetic graphene-based nanoparticles. (LOD, limits of detection; FID, flame ionization detector; UHPLC, ultra high performance liquid chromatography; SIM, selected ion monitoring; CNF, carbon nanofibers).

adsorbent	no of PAHs	sample	extraction technique	detection method	adsorbent mass	relative recoveries (%)	reusability absorbent	LOD	references
Fe_3_O_4_/GO	5	water	MSPE	HPLC-UV	40 mg	76.8−103.2	—	0.09−0.19 ng ml^−1^	[113]
G/Fe_3_O_4_@PT	5	seawater	MSPE	GC-FID	20 mg	83−107	17 times	0.009−0.020 mg l^−1^	[114]
Fe₃O₄@SiO₂@GO–PEA	10	water	MSPE	GC-FID	30 mg	71.7−106.7	20 times	0.005−0.1 μg l^−1^	[115]
m-G/CNF	5	water	MSPE	GC-FID	20 mg	95.5−99.9	6 times	0.004−0.03 ng ml^−1^	[116]
3D-IL@mGO	16	food	MSPE	GC-MS	50 mg	80.2−115	5 times	0.05−0.30 µg kg^−1^	[117]
Fe_3_O_4_/rGO	13	water	MSPE	GC-MS	10 mg	75.6−112.4	—	0.02−14.3 ng l^−1^	[118]
Fe_3_O_4_ @GO/PS	2	water	MSPE	GC-FID	15 mg	95.8−99.5	—	0.003−0.01 μg l^−1^	[110]
Fe_3_O_4_@GO-PANI	7	water	MSPE	GC-MS	2.5 mg	91.6 −114 (PAHs), 92.3−110 (N-PAHs)	15 times	0.04−0.05 ng ml^−1^ (PAHs) 0.01−0.11 ng ml^−1^ (N-PAHs)	[119]
FLG@TA@RSN-CN	1	water	—	UHPLC-FLD	5 mg l^−1^	98.82−99.95	3 times	—	[120]
3DG/Fe_3_O_4_	13	water	MSPE	GC-MS/SIM	10 mg	71−110	30 times	0.016−0.2 ng ml^−1^	[121]

**Figure 7.**
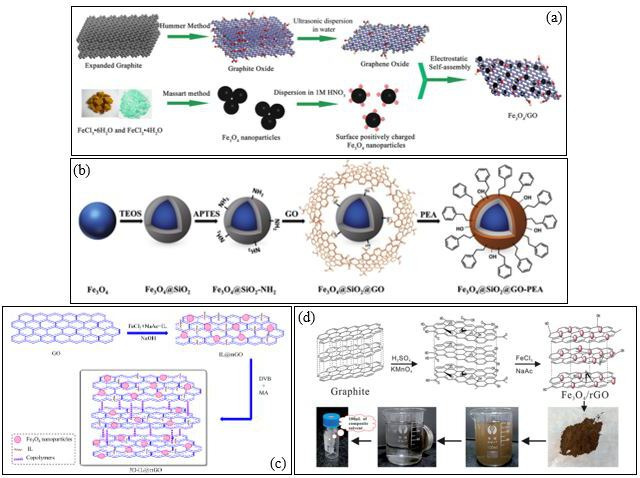
Synthesis method of the (a) Fe_3_O_4_/GO nanocomposite [113], (b) Fe₃O₄@SiO₂@GO–PEA microspheres [115], (c) 3D-IL@mGO [117], and (d) Fe_3_O_4_/rGO [118].

The implementation of magnetic graphene-based nanocomposites for the elimination of PAHs has attracted many researchers’ attention owing to their high adsorption efficiency, magnetic separation capabilities and reusability. Among these, Fe_3_O_4_/GO nanocomposites synthesized by Han *et al*. [113] via electrostatic self-assembly provide a simple and efficient method for PAH extraction (figure 7a). Negatively charged GO sheets interact electrostatically with positively charged Fe₃O₄ nanoparticles, forming a hybrid material with excellent adsorption properties. While this approach offers a rapid and environmentally friendly synthesis, its long-term reusability and adsorption capacity require further optimization. Building upon this concept, Mehdinia *et al*. [114] introduced polythiophene (PT) as a coating agent in graphene/ Fe_3_O_4_@polythiophene (G/ Fe_3_O_4_@PT) nanocomposites to enhance adsorption efficiency. The incorporation of PT improved the selectivity for PAHs while extending the adsorbent’s usability up to 17 cycles without notable degradation. Similarly, Mahpishanian *et al*. ([115]; figure 7b) functionalized Fe_3_O_4_@SiO₂@GO with phenylethylamine (PEA) to produce Fe_3_O_4_@SiO₂@GO-PEA, significantly increasing the compound hydrophobicity and π–π stacking interactions, leading to enhanced PAH extraction efficiency. The silica coating provided additional stability and dispersibility in aqueous solutions, a key improvement over Fe₃O₄/GO composites. Further advancements in the structural design of magnetic nanocomposites have been achieved through the incorporation of CNFs, ionic liquids and polymer modifications. Rezvani-Eivari *et al*. [116] synthesized magnetic graphene layers on branching CNFs (m-G/CNF), which offered high adsorption because of the complementary action of graphene sheets and CNFs, contributing to improving extraction efficiency. Around the same time, Zhang *et al*. ([117]; figure 7c) developed a three-dimensional ionic liquid functionalized MGO nanocomposite (3D-IL@mGO) using solvothermal and free-radical polymerization. The incorporation of an ionic liquid significantly enhanced mechanical stability and reusability, making it viable for at least five cycles with minimal efficiency loss.

Pang *et al*. [118] took a different approach by synthesizing magnetic reduced GO ( Fe_3_O_4_/rGO) using a solvothermal process (figure 7d). This adsorbent demonstrated a high adsorption capacity with minimal adsorbent dosage, requiring only 10 mg for 100 ml of water sample. Plus, its ability to fully release the absorbed compounds allowed for highly effective PAH recovery, confirming it as one of the most credible and precise methods. Amiri *et al*. [110] further optimized the surface characteristics of magnetic nanocomposites by incorporating polystyrene (PS) in GO- Fe_3_O_4_@PS, which improved extraction yield and detection sensitivity when analysed via GC-FID. Recent studies have focused on improving reusability and extraction efficiency. Manousi *et al*. [119] synthesized a Fe_3_O_4_@GO-polyaniline (PANI) composite that demonstrated high selectivity for PAHs and nitrated PAHs, maintaining efficiency for at least 15 applications without notable degradation. More recently, Vaz-Ramos *et al*. [120] synthesized FLG@TA@RSN-CN, a composite consisting of few-layer graphene coated with tannic acid and iron oxide raspberry-shaped nanostructures. This material achieved up to 99.95% removal of BaP, making it an effective adsorbent for PAH elimination. Finally, Sereshti *et al*. [121] introduced a three-dimensional graphene aerogel composite (3DG/ Fe_3_O_4_), which offered exceptional reusability for up to 30 cycles without efficiency loss, making a significant advancement in sustainable and cost-effective PAH removal methods. In comparison, Fe_3_O_4_/rGO and FLG@TA@RSN-CN demonstrated the highest adsorption efficiencies, whereas 3DG/ Fe_3_O_4_ exhibited superior reusability. The addition of polymer coatings such as PT, PS and PANI enhanced selectivity and extraction yield, while ionic liquid-modified nanocomposites like 3D-IL@mGO provided better mechanical stability. These advancements highlight the continuous evolution of magnetic graphene-based nanocomposites, offering promising solutions for the efficient, reusable and environmentally friendly extraction of PAHs from aqueous environments.

The advancements of adsorbent magnetic graphene-based nanocomposites have significantly enhanced the adsorption of PAHs from environmental water samples. Various functionalized materials listed here demonstrate exceptional adsorption capacities, high selectivity and efficient magnetic separation. These nanocomposites use strong π–π interactions, hydrophobic effects and improved dispersion properties, making them promising candidates for PAH removal. The integration of graphene-based materials with MNPs not only enhances adsorption efficiency but also facilitates eco-friendly, reusable and cost-effective water purification methods. Further research should focus on optimizing these new advancement substances for large-scale applications and expanding their use in the removal of other organic pollutants.

## 7. Mechanism of adsorption of polycyclic aromatic hydrocarbons

### 7.1. Mechanism adsorption of polycyclic aromatic hydrocarbons onto magnetic covalent organic frameworks

MCOFs have emerged as highly effective adsorbents for PAHs owing to their synergistic mechanism, including π–π stacking, hydrophobic interactions, hydrogen bonding and electrostatic attractions. Various MCOF-based materials exhibit different adsorption efficiencies based on their structural characteristics and functional modifications. Among the early developments, Li *et al*. [94] synthesized Fe_3_O_4_@COF-(TpBD), demonstrating superior efficiency of adsorption for PAHs in food samples owing to robust π–π stacking interactions and hydrophobic effects. Similarly, Wang & Chen [14] introduced COF-LZU1@PEI@ Fe_3_O_4_, which exhibited strong PAH adsorption capabilities attributed to its layered-sheet framework containing imine groups and benzene rings, further enhancing hydrophobic and π–π interactions. These materials successfully combined magnetic separation with COFs, enabling rapid and efficient extraction of trace analytes.

Further advancing MCOF performance, Wu *et al*. [29] developed Fe_3_O_4_@COF(Tp-NDA), incorporating a NAP ring structure that significantly enhanced affinity for PAHs through π–π stacking and hydrophobic interactions. This improvement in adsorption selectivity highlights the role of extended conjugated systems in increasing extraction efficiency. Similarly, Shi *et al*. [95] synthesized Fe_3_O_4_@COF(TpDA), which demonstrated remarkable efficiency in extracting 15 PAHs from culinary oils and grilled meats. The strong π–π stacking and hydrophobic interactions with targeted analytes further established its effectiveness for complex sample matrices. Meanwhile, Manousi *et al*. [119] introduced Fe_3_O_4_@GO-PANI, which exhibited exceptional performance in adsorbing PAHs and nitrated PAHs from water samples. The integration of PANI not only enhanced π–π interactions with PAHs but also improved stability and reusability, extending its usability across multiple extractions.

**Figure 8.**
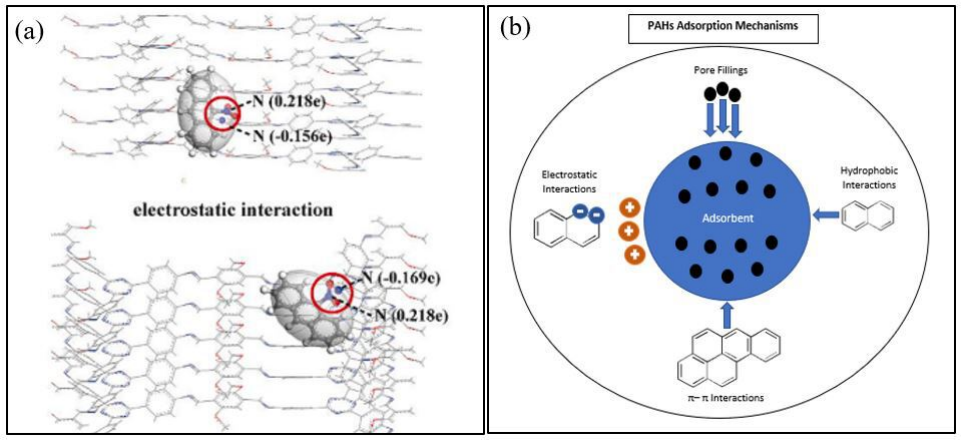
(a) N-ANT and TAPT-DMTA-COF electrostatic interaction [98]. (b) Overall adsorption mechanism of PAHs onto MCOFs.

A more structurally advanced approach was presented by Wang *et al*. [98] with Fe_3_O_4_@TAPT-DMTA-COF, featuring a grapevine architecture that outperformed conventional core-shell morphologies. The presence of electronegative and electropositive nitrogen atoms facilitated electrostatic interactions (figure 8a), while C–H…π stacking and π–π interactions further enhanced the adsorption of nitrated PAHs (N-PAHs). This material demonstrated superior sensitivity in detecting N-PAHs in environmental and dietary samples. Likewise, He *et al*. [93] synthesized a bouquet-shaped Fe_3_O_4_@TpPa-1 nanocomposite, integrating extensive π−π interactions with high nitrogen and oxygen content. The strong hydrophobic interactions, supported by hydrogen bonding and π−π stacking, resulted in efficient removal of organic compounds containing amino, benzene or hydroxyl groups.

Expanding the functional versatility of MCOFs, Hu *et al*. [96] introduced Fe_3_O_4_@COFeCOOH, a multifunctional carboxylate-functionalized COF composite. This material enabled concurrent adsorption of PAHs, TCs and TDs through mixed-mode solid-phase extraction (SPE). The presence of carboxyl functional groups enhanced π–π stacking, hydrogen bonding and ion exchange interactions, significantly increasing extraction efficiency. Notably, the enhanced design of the COFeBaP complex demonstrated nearly complete BaP aggregation on the conjugated COFeCOOH framework, indicating exceptionally high affinity for PAHs.

Overall, while all MCOF-based adsorbents rely on π–π stacking and hydrophobic interactions as their primary adsorption mechanisms, structural variations and functional modifications significantly influence their performance (figure 8b). Fe_3_O_4_@TAPT-DMTA-COF and Fe_3_O_4_@TpPa-1 exhibited advanced architectural designs that enhanced electrostatic and π–π interactions, leading to superior adsorption efficiencies. By contrast, Fe_3_O_4_@COFeCOOH showcased remarkable versatility by targeting multiple analytes through mixed-mode interactions. Meanwhile, Fe_3_O_4_@COF(Tp-NDA) and Fe_3_O_4_@COF(TpDA) demonstrated high specificity for PAHs, making them ideal for food and environmental sample analysis. The incorporation of conductive polymers like PANI and functional groups such as carboxyl further optimized extraction performance and reusability. These advancements highlight the continuous evolution of MCOFs as highly efficient, selective and reusable adsorbents for PAH extraction and environmental remediation.

### 7.2. Mechanism of adsorption of polycyclic aromatic hydrocarbons onto magnetic graphene nanoparticles

**Figure 9.**
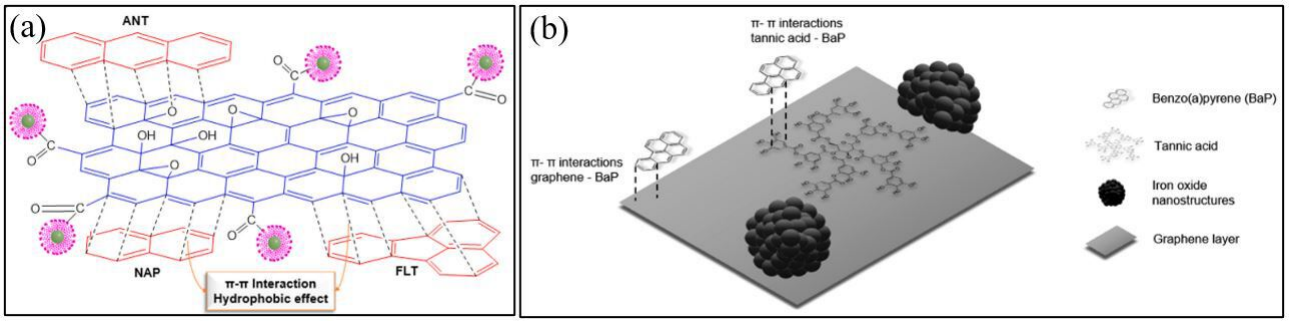
Mechanism of adsorption. (a) PAHs into G-mCS/GO [75] and (b) composite nanomaterials (CNs) and BaP [120].

Magnetic graphene nanoparticles have demonstrated excellent adsorption efficiency for PAHs because of their extensive delocalized π–electron system, hydrophobic nature and functionalized active sites. Various studies have shown that different functional modifications and synthesis methods significantly influence the adsorption efficiency of PAHs by enhancing π–π stacking interactions, hydrophobic effects and other secondary interactions such as electrostatic attraction, hydrogen bonding and ion exchange. Queiroz *et al*. ([67]; figure 9a) synthesized two composites, G-mCS/GO and C-mCS/GO, using green and conventional methods, respectively. The presence of oxygen-containing functional groups such as C=O and O–H contributed to strong π–π interactions with PAH molecules. Among the PAHs tested, NAP exhibited superior stability and adsorption affinity, highlighting the importance of molecular size and π–electron interactions in adsorption efficiency. The hydrophobic effect further facilitated PAH adsorption, making these composites effective adsorbents for wastewater treatment.

Similarly, Mahpishanian *et al*. [115] developed Fe_3_O_4_@SiO₂@GO–PEA, a superparamagnetic nanocomposite that combined the hydrophobic properties of PEA with the extensive surface area of GO. The introduction of PEA significantly improved π–π stacking interactions with PAHs, with oxygen-containing functional groups on GO enhancing dispersibility in aqueous solutions and ensuring efficient adsorption. This combination resulted in superior sensitivity, chemical stability and analytical performance, making it highly effective for preconcentration of PAHs using MSPE prior to GC-FID analysis. Comparatively, Mehdinia *et al*. [114] synthesized G/Fe_3_O_4_@PT, a non-polar and hydrophobic adsorbent with a strong affinity for carbon-based rings and hydrophobic structures. The extensive delocalized π–electron system played a crucial role in forming robust π–π stacking interactions with PAHs. Additionally, cation–π bonding facilitated the adsorption of metal ions, broadening its applicability beyond PAHs to include inorganic contaminants.

Advancing the adsorption performance, Vaz-Ramos *et al*. [120] developed FLG@TA@RSN, which demonstrated exceptional efficacy in adsorbing BaP through π–π interactions between BaP and graphene (figure 9b). The presence of tannic acid further enhanced these interactions by providing additional aromatic rings, contributing to improved BaP adsorption. This highlights the role of functionalized aromatic groups in increasing adsorption efficiency. In a similar approach, Sereshti *et al*. [121] grafted phenyl rings of PEA onto GO nanosheets, creating a delocalized electron system that significantly improved PAH adsorption selectivity. The introduction of 3DG/Fe₃O₄ further strengthened π–π stacking interactions with aromatic ring-containing molecules, demonstrating a high affinity for PAHs. Likewise, Amiri *et al*. [110] have successfully synthesized GO-Fe_3_O_4_@PS, where PS coating enhanced surface-active sites, promoting both hydrophobic interactions and π–π stacking. The increased surface area further optimized analyte exposure, significantly improving the adsorption capability of the material.

Overall, while all magnetic graphene-based adsorbents rely primarily on π–π stacking interactions and hydrophobic effects, their performance varies based on functionalization and structural modifications. Composites such as Fe_3_O_4_@SiO₂@GO–PEA and G/Fe_3_O_4_@PT exhibit enhanced π–π interactions and hydrophobicity, making them highly effective in aqueous environments. The incorporation of aromatic functional groups, as seen in FLG@TA@RSN and 3DG/Fe_3_O_4_, further improves selectivity and adsorption efficiency. By contrast, materials like GO-Fe_3_O_4_@PS use increased surface-active sites to optimize adsorption capacity. The diverse mechanisms of interaction, including cation–π bonding and electrostatic attractions, highlight the versatility of graphene-based adsorbents in environmental remediation. By comparing these materials, it is proved that strategic functionalization and structural design are important elements in maximizing PAH adsorption efficiency, making magnetic graphene-based nanocomposites highly promising for pollutant removal and analytical applications.

## 8. Covalent organic framework synthesis with deep eutectic solvents

COFs have been shown to be an effective adsorbent owing to their good porosity, chemical stability and thermal resilience. However, their conventional synthesis methods, including solvothermal, mechanochemical and ion-thermal approaches, often involve substantial energy consumption, prolonged reaction durations (typically 72−168 h) and the usage of harmful organic solvents [16,122]. Solvothermal synthesis, the most employed method, involves heating precursor materials in a solvent mixture under self-generated pressure, where the choice of solvent is critical but often problematic because of toxicity, flammability and instability during the process [16]. Moreover, achieving thermodynamic equilibrium is essential in these conventional methods to facilitate self-correction of defects during crystallization, adding to the complexity of the process. These constraints have prompted the exploration of more sustainable synthesis techniques, with DESs emerging as promising sustainable alternatives.

DESs, a novel class of ionic liquids, were developed to overcome the constraints of traditional organic solvents. Unlike ionic liquids, DESs are synthesized by combining a hydrogen bond donor, such as urea or carboxylic acids, with a salt like choline chloride (ChCl) [123,124]. This straightforward one-step synthesis eliminates the need for post-synthesis purification or energy-intensive operations. DESs exhibit excellent properties of having low vapour pressure, high thermal stability, excellent conductivity and reusability, making them attractive alternatives for COF synthesis [17,125]. Furthermore, their biobased and recyclable components significantly reduce toxicological and ecological footprints compared with conventional organic solvents [126]. The ability of DESs to promote sorption performance by hydrogen bonding to material surfaces [127] further enhances their use in COF synthesis.

**Figure 10.**
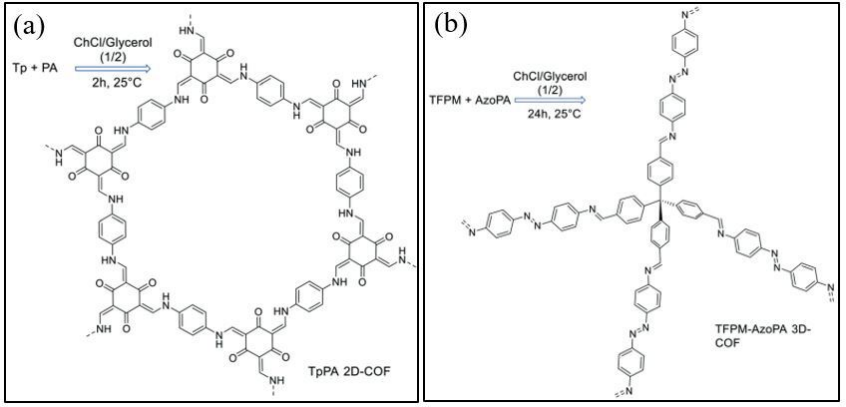
Mechanism reaction synthesizing (a) two-dimensional (including keto-enamine, azine and hydrazone-linked COFs) and (*b*) imine-linked three-dimensional COFs using DESs [122].

Recent studies have demonstrated the effectiveness of DESs in facilitating COF synthesis under milder conditions, significantly improving reaction efficiency and sustainability. Qiu *et al*. [122] pioneered a method for synthesizing both two-dimensional (keto-enamine, azine and hydrazone-linked) and three-dimensional (imine-linked) COFs using DESs at ambient conditions (figure 10). This approach yielded highly crystalline COFs with significant surface areas in under 2 h, a considerable improvement over conventional polycondensation methods in ionic liquids, which require approximately 12 h at elevated temperatures. Notably, this method enabled the production of a novel azo-based three-dimensional COF with a hierarchical pore structure, which was unattainable through traditional solvothermal techniques. This advancement highlights the potential of DESs in expanding the scope of COF structures.

Building on this progress, Dong *et al*. [128] synthesized a carboxyl-functionalized COF (TpPa-COOH) for the first time using a DES consisting of tetrabutylammonium bromide and imidazole (Bu₄NBr/Im). This method eliminated the need for volatile organic solvents, using the low vapour pressure of DESs to enable synthesis at ambient pressure. The resulting COF revealed a notable adsorption potential of 1135 mg g^−1^ for organic dyes, driven by π–π interactions, electrostatic forces and hydrogen bonding. Similarly, Liu *et al*. [20] developed a green synthesis approach for imine-linked COFs using DESs as a solvent system at room temperature and normal pressure. Their COFs demonstrated high crystallinity, extensive surface areas (approx. 1300 m² g^−1^), and mesoporous architectures, achieving nearly 100% dye removal in just 3 min. Additionally, their study assessed the thermal stability of DESs and the catalytic role of acetic acid in COF synthesis, revealing that the synthesized COFs maintained their crystallinity after multiple reuse cycles.

Overall, the transition from conventional solvothermal methods to DES-based synthesis represents a significant advancement in COF fabrication. While solvothermal techniques require high temperatures, extended reaction times and hazardous solvents, DES-based synthesis enables rapid COF formation under mild conditions with lower environmental impact. The ability to conduct reactions at reduced temperatures, in open-air vessels and within shorter time frames underscores the advantages of DESs over traditional methods. Furthermore, DES-based synthesis has facilitated the development of COFs with novel structures and diverse linkages that were previously challenging to obtain using conventional approaches. Despite these promising advancements, the employment of DESs in the synthesis process and functionalized with COF remains in its early stages, necessitating further research to fully explore their potential benefits, optimize reaction conditions and expand their practical applications.

## 9. Adsorbent modified with deep eutectic solvent for the adsorption of polycyclic aromatic hydrocarbons

The implementation of sustainable DES in the modification and fabrication of adsorbent materials has recently gained interest from researchers. These adsorption materials are essential for water treatment primarily by facilitating the degradation or adsorption of pollutants. As concerns over environmental sustainability grow, these advanced materials are emerging as promising alternatives to traditional water treatment methods [129]. Their potential to enhance efficiency while reducing harmful chemical usage makes them a key focus in the development of greener and more sustainable remediation technologies [130]. Further research is being conducted to optimize their properties, improve performance and expand their practical applications in large-scale environmental clean-up efforts. Thus, in this review, we listed the adsorbents modified with DES for enhancing the adsorption of PAH in table 4 . However, the research on adsorbents modified with DES for the adsorption of PAHs is still in its preliminary phase, and further studies are essential to fully discover its attributes and applications.

**Table 4.** Adsorbent modified with DES for the adsorption of PAH. (DLLME, dispersive liquid–liquid microextraction; MD–µSPE, magnetic dispersive micro-solid phase extraction; SPME, solid-phase microextraction; AALLME, air-assisted liquid–liquid microextraction; LOD, limits of detection.)

adsorbent	no of PAHs	samples	extraction technique	detection method	relative recoveries (%)	LOD	DES composition	references
pAS-COOH-GO	8	lake water	SPE	HPLC-UV	90.8−107.5	0.02−0.38 ng ml^−1^	ChCl:ethanediol EG 1:3	[131]
NADESs@Fe_3_O_4_-DES	14	coffee	microextraction	GC-MS HPLC-FLD	91.3−121	0.31−5.9 ng l^−1^	menthol:borneol:camphor 5:1:4	[132]
Fe3O4@TEOS–DES	16	meat	DLLME	GC-MS	73−92	0.029−0.082 ng ml^−1^	PChCl:menthol:decanoic acid 1:1:1	[133]
Toner@Aliquat 336-DES ferrofluid	16	saliva and urine	AALLME	GC-MS	61−84 (saliva), 305−420 (urine)	0.018−0.063 ng ml^−1^	ChCl:stearic acid 1:2	[133]
ChCl-decanoic acid DES@Fe nanoparticles	16	mascara	MD–µSPE	GC-FID	80−95	0.33 – 0.57 µg l^−1^	ChC:ldecanoic acid 1:2	[134]
MOF-DES/MIPs	4	soil, vegetables, water, and wastewater	SPME	GC-FID	94.3−106.1	0.015 to 0.019 μg l^−1^	ChCl:glycerol 2:1	[135]

The adsorption of PAHs has been significantly enhanced through the functionalization of adsorbents using DESs, offering an environmentally green replacement for conventional approaches. Various DES-based modifications are conducted to improve adsorption efficiency, selectivity and stability [136]. Natural deep eutectic solvents (NADESs), a subclass of DESs, have been increasingly recognized as viable substitutes for ionic liquids. Fan *et al*. [132] developed a rapid and straightforward microextraction technique using NADES modified with nanoferrofluid for PAH detection in complex matrices. The ternary NADES system, comprising menthol, borneol and camphor, successfully stabilized Fe_3_O_4_ nanoparticles, forming a stable ferrofluid. This method demonstrated significant sensitivity with low limits of detection (0.31−5.9 ng l^−1^) and high recovery rates (91.3−121%), indicating its potential for sustainable PAH measurement in food samples.

The modification of GO using DESs has also been explored for PAH adsorption. Shen *et al*. [131] synthesized a DES-based monolithic chip incorporating GO for SPE of PAHs from aqueous samples. The high viscosity of DES facilitated uniform GO dispersion, preventing aggregation. The carboxylated GO, functionalized with p-aminostyrene and integrated into a poly (butyl methacrylate-co-ethylene glycol dimethyl methacrylate) monolithic chip, effectively concentrated aromatic compounds. Mehrabi *et al*. [137] further demonstrated the potential of DES-functionalized GO for adsorption by employing a ChCl-urea DES to conjugate Fe₃O₄ nanoparticles onto GO nanosheets. The resulting DES/GO-Fe_3_O_4_ nanohybrids exhibited superior adsorption efficiency for organic contaminants in water compared with unmodified GO or Fe_3_O_4_ alone, highlighting the enhanced adsorption capabilities conferred by DES functionalization.

Magnetic dispersive techniques using DESs have also gained attention for PAH extraction and adsorption. Jouyban *et al*. [138] introduced an ultrasonic-assisted ferrofluid-based dispersive liquid–liquid microextraction coupled with microwave-assisted counter-current extraction for PAH detection in grilled meat samples. The stable ferrofluid, created from Fe_3_O_4_@TEOS nanoparticles dispersed in a ternary deep eutectic solvent (PChCl, menthol and octanoic acid), demonstrated low detection limits and high extraction recoveries. Additionally, Jouyban *et al*. [138] developed a novel ferrofluid-based extraction method using toner powder and a water-immiscible DES (ChCl:stearic acid) for PAH detection in urine and saliva samples from smokers. The addition of Aliquat 336 as a surfactant prevented agglomeration, thereby improving extraction efficiency. Similarly, Dilmaghani *et al*. [134] employed a ChCl-decanoic acid DES coating for iron nanoparticles derived from Fe(CO)_5_ in the analysis of PAHs in mascara samples via magnetic dispersive micro-solid phase extraction. This technique demonstrated excellent linearity, low detection limits (0.33−0.57 µg l^−1^) and high extraction recoveries, further emphasizing the potential of DES-modified magnetic adsorbents for PAH removal.

Recent advancements have shifted focus from hydrophilic magnetic DESs, which primarily target polar chemicals, to magnetic hydrophobic deep eutectic solvents (MHDESs) that efficiently extract non-polar PAHs from aqueous solutions. Farooq *et al*. [139] compared two MHDES systems, one based on tetraoctylammonium bromide and octanoic acid and another based on trioctylphosphine oxide and octanoic acid, concluding that the latter exhibited superior extraction efficacy. This shift towards hydrophobic DESs represents a significant breakthrough in enhancing the adsorption of PAHs, particularly for non-polar contaminants.

Further expanding DES applications, Mirzajani & Dianati [135] introduced a solid-phase microextraction fibre incorporating MOFs, DESs and molecularly imprinted polymers (MIPs) (MOF-DES/MIPs). This hybrid material significantly improved fibre capacity, selectivity and chemical stability, allowing efficient PAH extraction from environmental matrices such as soil, plants, water and wastewater. The technique offered practical advantages, including cost-effective eutectic solvents, a straightforward experimental protocol, superior detection limits, linearity and reproducibility.

Collectively, these studies underscore the growing role of DES-functionalized materials in PAH adsorption, demonstrating their effectiveness in modifying GO, ferrofluids and MIPs. The transition from conventional solvents to DESs has led to more sustainable and efficient adsorption processes. While the use of DESs in PAH adsorption is still evolving, ongoing research into novel DES-based methodologies is expected to yield further improvements in environmental remediation and food safety applications.

Several empirical studies have substantiated the practical use of MCOFs and DES-functionalized nanomaterials in environmental and food safety contexts, reinforcing their potential for large-scale deployment. For instance, the application of Fe_3_O_4_@COFeCOOH [96], a multifunctional MCOF composite for simultaneous removal of PAHs, tetracyclines and triphenylmethane dyes from aqueous matrices, exemplifies the versatility of these adsorbents in addressing multi-contaminant pollution. Its demonstrated stability and reusability over 15 cycles indicate economic and operational viability for industrial wastewater treatment. Likewise, the integration of DES-functionalized adsorbents in complex food matrices, such as grilled meats and edible oils, illustrates their efficacy in trace-level PAH detection through MSPE techniques. One such example is the Fe_3_O_4_@COF(TpDA) composite [95], which achieved high recovery rates and low detection limits within minimal contact times. In the domain of personal care product analysis, DES-coated MNPs [135] have successfully extracted PAHs from cosmetics such as mascara, showcasing the adaptability of these systems in viscous, oil-based matrices. Furthermore, *in situ* environmental monitoring has been enhanced using DES-based MOF-imprinted polymer fibres, allowing for sensitive, selective and reproducible PAH extraction in heterogeneous samples like soil, wastewater and vegetation.

Several studies have also demonstrated the successful synthesis of MCOFs using DES for the adsorption of various pollutants beyond PAHs. Notably, a novel MSPE method coupled with GC-MS/MS was developed for the sensitive and selective detection of 16 pyrethroid pesticides [140]. This green synthesis strategy, which employed DESs, significantly reduced the reaction time to just 3 h, much shorter than traditional approaches, while avoiding the use of toxic organic solvents. The resulting MCOFs (urea-MTpBD) exhibited a high surface area, strong magnetic properties and efficient adsorption performance through π–π stacking and hydrophobic interactions. The method demonstrated low detection limits, good recovery rates and high precision, and was successfully applied to real-world samples such as tobacco, fruits and vegetables. Furthermore, the MCOFs showed excellent reusability, underscoring their promise for cost-effective and environmentally sustainable pesticide analysis. An MCOF-DES was developed as an efficient adsorbent for magnetic dispersive micro solid-phase extraction (MDMSPE) of trace copper ions (Cu²^+^) in medicinal plants and environmental samples[141]. This advanced material addressed key limitations of traditional MDMSPE adsorbents by offering enhanced selectivity, anti-interference capability and adsorption capacity. This method’s robustness and its potential act as a selective and sustainable strategy for trace heavy metal analysis in complex matrices.

These case studies collectively validate the performance, selectivity and reusability of MCOF- and DES-based systems, highlighting their readiness for regulatory validation and commercial translation.

## 10. Interdisciplinary perspectives, challenges and future research directions

### 10.1. Interdisciplinary connections

The development of graphene-based MCOFs and DES-functionalized adsorbents for the remediation of PAHs represents a confluence of several scientific domains, most notably materials science, environmental engineering, chemistry and nanotechnology. From a materials science perspective, the structural design of COFs is pivotal in determining their adsorption capabilities. The structural design of COFs critically influences their adsorption performance. Incorporation of DESs, especially into MCOFs, enhances selectivity, stability and efficiency in removing PAHs [131,137,142]. NADESs offer green alternatives to ionic liquids and have been used to stabilize nanomaterials like Fe₃O₄ and GO, improving dispersion and preventing aggregation. Magnetic DES-based techniques have shown high recovery and low detection limits for PAHs in complex matrices such as grilled meats, cosmetics and biofluids [134,138]. Recent work has also shifted towards MHDESs for more effective extraction of non-polar PAHs [139]. Hybrid materials combining MOFs, DESs and MIPs have demonstrated enhanced selectivity and durability in environmental sample analysis [135]. Beyond PAHs, DES-based MCOFs have been employed for multi-contaminant removal including antibiotics, dyes, pesticides and heavy metals, showcasing their versatility, reusability and potential for industrial applications [139].

Nanotechnology contributes significantly to the performance of these adsorbents. Nanotechnology has enabled the development of various advanced methods such as nano-adsorbents, nanofiltration systems, nanophotocatalysts, MNPs and nanosensors for applications in water and wastewater treatment, air purification and pollutant detection. Owing to its effectiveness in removing, controlling and preventing the spread of environmental contaminants, nanotechnology is increasingly recognized as a form of green technology and a powerful tool for advancing SDGs [143]. For example, a nanocomposite material consisting of three-dimensional graphene aerogel and iron oxide nanoparticles (3DG/Fe_3_O_4_) was created and used for the MSPE of 13 PAH compounds, showcasing high adsorption capacity and ease of separation from sample solutions. Moreover, a green magnetic composite based on chitosan and GO was synthesized using water hyacinth extract and proanthocyanidin, effectively adsorbing NAP from wastewater with high efficiency and reusability.

In the realm of environmental engineering, these advanced adsorbents offer promising solutions for pollutant removal. A study reported the fabrication of a magnetic graphene nanocomposite (Fe_3_O_4_/rGO) applied to the MSPE of PAHs in environmental water samples, demonstrating high adsorption capacity and efficient desorption [121]. Furthermore, a multifunctional sorbent based on core-shell magnetic carboxylate-functionalized COF composites (Fe_3_O_4_@COF-COOH) [96] was developed for the simultaneous adsorption of PAHs and other organic pollutants from environmental water, highlighting the potential for comprehensive water purification strategies.

These interdisciplinary studies underscore the multifaceted nature of magnetic graphene COFs and DES-functionalized adsorbents, emphasizing their relevance across various scientific domains.

### 10.2. Challenges and limitations

Despite significant advances, several critical challenges must be addressed to accelerate the real-world implementation of MCOFs and DES-functionalized adsorbents. Technically, the synthesis of MCOFs often involves multiple steps, including COF crystallization and magnetic nanoparticle incorporation, which may be difficult to scale without compromising crystallinity, surface area or magnetic properties. While DESs offer an environmentally benign alternative to traditional solvents, their long-term stability, compatibility with diverse adsorbent matrices and environmental fate remain underexplored. Economic constraints also play a role, as the cost of high-purity precursors, especially for specialty DESs or heteroatom-rich COFs, may hinder commercialization unless cost-effective synthesis protocols are developed. From an environmental standpoint, the deployment of nano-adsorbents raises concerns about their persistence and potential ecotoxicity, especially in aquatic ecosystems where nanomaterials may interact unpredictably with biota. Additionally, there is limited understanding of adsorbent degradation pathways, regeneration by-products and their impacts on overall system sustainability. Regulatory uncertainty and the lack of standardized performance metrics for emerging adsorbent technologies further complicate field adoption. To overcome these limitations, comprehensive techno-economic analyses, ecotoxicological assessments and pilot-scale validation studies must be integrated into ongoing research initiatives.

### 10.3. Future research directions

To fully harness the potential of MCOFs and DES-modified adsorbents for PAH remediation, several key areas demand more focused and in-depth exploration. First, the direct synthesis of MCOFs using DESs remains relatively limited. This represents a significant gap in the current research landscape and should be prioritized as a promising direction for future development, given its potential to streamline fabrication, reduce solvent toxicity and enhance environmental sustainability. Second, advancements in synthesis methodologies, especially those that operate under ambient pressure and room temperature, should be emphasized to improve scalability and lower energy demands. Using DESs in these processes can further support green chemistry initiatives. The design and application of bio-derived DESs with tuneable functional groups and improved hydrophobicity could significantly enhance the affinity of adsorbents towards non-polar PAHs. Third, integrating MCOFs with biodegradable or biocompatible materials such as chitosan, cellulose or lignin-based frameworks could improve the environmental compatibility of adsorbents and facilitate recovery and reuse in eco-friendly water treatment systems. In parallel, mechanistic studies using advanced spectroscopic, microscopic and computational tools are needed to elucidate adsorption pathways and dynamics under realistic environmental conditions, including varying pH, ionic strength and the presence of co-contaminants. Fourth, long-term field trials and pilot-scale demonstrations are essential to validate the practical performance, reusability and life-cycle impact of these materials. Moreover, the development of stimuli-responsive or adaptive adsorbents, capable of modulating their affinity in response to environmental triggers (e.g. pH or redox changes), could offer a next-generation solution for targeted and efficient remediation. Finally, establishing a robust interdisciplinary framework that brings together materials scientists, environmental chemists, engineers, regulatory authorities and industry stakeholders is critical. Such collaboration will enable the development of standardized testing protocols, ensure knowledge transfer and align emerging technologies with regulatory frameworks and societal expectations.

## 11. Conclusions

This review highlights the advancement and application of graphene-based MCOFs and DES-modified adsorbents as an efficient removal material for PAHs. Compared with traditional adsorbent materials, which often encounter limitations such as low adsorption capacity, secondary pollution and complex regeneration processes, MCOFs present as a newly developed type of functional material. These advanced materials combine the high surface area and adjustable porosity of COFs with the durability, electrical conductivity and their ability to functionalize with magnetic properties such as graphene-based nanocomposites. This unique combination makes MCOFs a highly efficient and versatile option for various applications, including environmental remediation and pollutant removal. The introduction of DESs as a green modification strategy to further optimize adsorption efficiency by lowering reaction temperature reduces synthesis time and eliminates the usage of hazardous organic solvents, addressing key challenges in water treatment.

The novelty of this review is in its dual focus on material innovation and sustainable green chemistry. By systematically discussing the role of graphene-based MCOFs in PAH adsorption, this article highlights the critical role of adsorption mechanisms involving π–π stacking interactions, hydrophobic effects and enhanced structural stability in improving adsorption efficiency. Moreover, this work introduces DESs as an advanced green chemistry approach over traditional techniques using harmful organic solvents in COF synthesis. This unique combination of MCOFs and DES-modified adsorbents underscores their potential to be the next-generation materials for large-scale environmental remediation applications.

Beyond the material-specific insights, this review underscores that the advancement of highly efficient, reusable and cost-effective adsorbents aligns with the objectives of SDGs 6, 7 and 14, ensuring clean water and sanitation, promoting affordable and clean energy and protecting aquatic ecosystems. The ability of MCOFs and DES-modified materials to address persistent organic pollutants makes them strong candidates for future applications in industrial wastewater treatment, oil spill remediation and emerging pollutant removal.

Moving forward, future research should explore the large-scale synthesis and industrial application of these materials, with a focus on enhancing their recyclability, stability under real-world situations and cost-effectiveness, specifically focusing on environmental applications. In addition, further studies should be conducted to investigate the long-term environmental impact of these nanomaterials and their potential integration with existing water treatment methods.

## Data Availability

The authors are unable or have chosen not to specify which data has been used.
